# Supplementary material for: Availability and use of personal protective equipment in low- and middle-income countries during the COVID-19 pandemic
Source: PLoS One. 2023 Jul 17;18(7):e0288465. doi: 10.1371/journal.pone.0288465 (PMC10351736; doi:10.1371/journal.pone.0288465)
Supplement: S1 Table — (DOCX) [file pone.0288465.s001.docx]

**S1 Table Availability of PPE at the health facilities over rounds**

| **Round** | **Country** | **Gown** | **Gloves** | **Goggles** | **Face shields** | **N95/FFP2** | **Medical masks** |
| --- | --- | --- | --- | --- | --- | --- | --- |
| Jun-21 | Bangladesh | 49% | 56% | 41% | 42% | 20% | 65% |
| Jul-21 | Bangladesh | 51% | 55% | 42% | 38% | 25% | 63% |
| Jul-21 | Burkina Faso | 88% | 94% | 68% | 84% | 51% | 66% |
| Aug-21 | Burkina Faso | 94% | 97% | 79% | 91% | 59% | 55% |
| Jun-21 | Guatemala | 79% | 93% | 82% | 79% | 72% | 89% |
| Jun-21 | Guinea | 69% | 73% | 56% | 57% | 15% | 25% |
| Jul-21 | Guinea | 64% | 63% | 47% | 52% | 10% | 31% |
| Nov-20 | Liberia | 95% | 82% | 88% | 91% | 78% | 71% |
| Feb-21 | Liberia | 92% | 71% | 80% | 85% | 67% | 66% |
| Jun-21 | Liberia | 92% | 94% | 84% | 93% | 76% | 70% |
| Jul-21 | Liberia | 97% | 97% | 85% | 91% | 80% | 78% |
| Feb-21 | Malawi | 65% | 98% | 68% | 95% | 67% | 100% |
| May-21 | Malawi | 61% | 96% | 67% | 85% | 74% | 98% |
| Jun-21 | Malawi | 68% | 95% | 73% | 88% | 74% | 97% |
| Feb-21 | Nigeria | 61% | 88% | 43% | 49% | 55% | 72% |
| Mar-21 | Nigeria | 61% | 93% | 46% | 46% | 59% | 76% |
| Apr-21 | Nigeria | 61% | 95% | 45% | 42% | 57% | 80% |
| May-21 | Nigeria | 70% | 95% | 45% | 50% | 61% | 80% |
