# Supplementary material for: Availability and use of personal protective equipment in low- and middle-income countries during the COVID-19 pandemic
Source: PLoS One. 2023 Jul 17;18(7):e0288465. doi: 10.1371/journal.pone.0288465 (PMC10351736; doi:10.1371/journal.pone.0288465)
Supplement: S2 Table — (DOCX) [file pone.0288465.s002.docx]

**S3 Table. Response rate to the health facility phone survey for the round of the study**

| **Country** | **Round** | **# of health facilities selected** | **# of health facilities interviewed** | **# of health facilities who cannot be reached or replaced** | **Response rate** |
| --- | --- | --- | --- | --- | --- |
| Bangladesh | Jul-21 | 300 | 291 | 9 | 98% |
| Burkina Faso | Aug-21 | 159 | 159 | 0 | 100% |
| Guatemala | Jun-21 | 255 | 239 | 16 | 94% |
| Guinea | Jul-21 | 160 | 156 | 4 | 98% |
| Liberia | Jul-21 | 122 | 116 | 6 | 97% |
| Malawi | Jun-21 | 204 | 192 | 12 | 94% |
| Nigeria | May-21 | 421 | 401 | 20 | 95% |
