# Supplementary material for: Availability and use of personal protective equipment in low- and middle-income countries during the COVID-19 pandemic
Source: PLoS One. 2023 Jul 17;18(7):e0288465. doi: 10.1371/journal.pone.0288465 (PMC10351736; doi:10.1371/journal.pone.0288465)
Supplement: S3 Table — (DOCX) [file pone.0288465.s003.docx]

**S4 Table Availability of PPE before the COVID-19 pandemic in Burkina Faso and Guinea**

| **Country** | **Gown** | **Gloves** | **Goggles** | **Face shields** | **N95/FFP2** | **Medical masks** |
| --- | --- | --- | --- | --- | --- | --- |
| Burkina Faso | 81% | 98% | 60% | 40% | 12% | 25% |
| Guinea | 55% | 64% | 41% | 43% | 10% | 31% |
