# Supplementary material for: Availability and use of personal protective equipment in low- and middle-income countries during the COVID-19 pandemic
Source: PLoS One. 2023 Jul 17;18(7):e0288465. doi: 10.1371/journal.pone.0288465 (PMC10351736; doi:10.1371/journal.pone.0288465)
Supplement: Attachment — Submitted filename: Response to Reviewers.docx [file pone.0288465.s005.docx]

To the editorial board of PLOS ONE,

Thank you for the invitation to respond to your and the reviewers’ comments and revise our manuscript. The comments were very helpful in improving the writing and better presenting our methodology and communicate our findings.

We are excited to submit a revised and improved manuscript for your review. Please find below a point-by-point response to the review comments. For easier reference, the received comments are boldfaced.

Sincerely,

Salomé Drouard

## **Editor’s comments**

**1. Please ensure that your manuscript meets PLOS ONE's style requirements, including those for file naming. The PLOS ONE style templates can be found at.**

Response: Thank you. We have revised the manuscript according to the PLOS ONE’s guidelines.

**2. Please provide additional details regarding participant consent. In the ethics statement in the Methods and online submission information, please ensure that you have specified what type you obtained (for instance, written or verbal, and if verbal, how it was documented and witnessed). If your study included minors, state whether you obtained consent from parents or guardians. If the need for consent was waived by the ethics committee, please include this information.**

Response: Thank you for the clarification, we added the following paragraph in the method section.
In the data collection paragraph: *“Survey respondents generally included facility officer in-charges, but in some cases other respondents, like facility administrators, were better suited to answer modules within the survey.*

In the ethical approval paragraph*: “Survey participation was voluntary and verbal consent was received from all respondents.”*

**3. Your ethics statement should only appear in the Methods section of your manuscript. If your ethics statement is written in any section besides the Methods, please move it to the Methods section and delete it from any other section. Please ensure that your ethics statement is included in your manuscript, as the ethics statement entered into the online submission form will not be published alongside your manuscript.**

Response: We added the following paragraph in the ethical approval paragraph of the method section.

*“The study was requested, reviewed, and approved by a director-level official in each Ministry of Health and was exempted from human subjects research as public health practice in every country except Burkina Faso. In Burkina Faso, ethical approval was received from the ethics committee of the local author’s institute.”*

**4. Please include a caption for figure 1.**

Response: Thank you. The caption was added to the main text with a legend.

**5. Please include captions for your Supporting Information files at the end of your manuscript, and update any in-text citations to match accordingly. Please see our Supporting Information guidelines for more information: http://journals.plos.org/plosone/s/supporting-information.**

Response: The captions were added, and the supporting information file updated according to the guidelines.

**6. Please review your reference list to ensure that it is complete and correct. If you have cited papers that have been retracted, please include the rationale for doing so in the manuscript text, or remove these references and replace them with relevant current references. Any changes to the reference list should be mentioned in the rebuttal letter that accompanies your revised manuscript. If you need to cite a retracted article, indicate the article’s retracted status in the References list and also include a citation and full reference for the retraction notice.**

Response: Thank you for your careful review and we apologize for this discrepancy due to an error in importing the bibliography from Mendeley. The following references were added in this order.

1. Cancedda C, Davis SM, DIerberg KL, Lascher J, Kelly JD, Barrie MB, et al. Strengthening Health Systems While Responding to a Health Crisis: Lessons Learned by a Nongovernmental Organization During the Ebola Virus Disease Epidemic in Sierra Leone. J Infect Dis. 2016;214: S153–S163. doi:10.1093/INFDIS/JIW345
2. Reddy SC, Valderrama AL, Kuhar DT. Improving the Use of Personal Protective Equipment: Applying Lessons Learned. Clinical Infectious Diseases. 2019;69: S165–S170. doi:10.1093/CID/CIZ619
3. Keep health workers safe to keep patients safe: WHO. [cited 28 Nov 2021]. Available: <https://www.who.int/news/item/17-09-2020-keep-health-workers-safe-to-keep-patients-safe-who>
4. Second round of the national pulse survey on continuity of essential health services during the COVID-19 pandemic. [cited 28 Nov 2021]. Available: <https://www.who.int/publications/i/item/WHO-2019-nCoV-EHS-continuity-survey-2021.1>
5. Kazungu J, Munge K, Werner K, Risko N, Vecino-Ortiz AI, Were V. Examining the cost-effectiveness of personal protective equipment for formal healthcare workers in Kenya during the COVID-19 pandemic. BMC Health Serv Res. 2021;21: 1–7. doi:10.1186/S12913-021-07015-W/FIGURES/5

**Reviewer 1’s comments**

**1. The English used is in the present tense, while grammatically all manuscripts must be in the past tense, particuarly when it is a retrospective analysis.**

Response: Thank you for pointing this out. The manuscript was re-written in past tense.

**2. This is a survey which does not state how many times a telephone call or a contact was made and how many times was the response in the affirmative or negative.**

Response: Thank you for this comment. We added the following extract to the data collection paragraph in the method section and the following table to the supporting information:

*Three attempts were made to reach each facility, and interview times were scheduled in advance to minimize burden on the respondents. In case of non-response, a replacement facility of the same facility level in the same province was randomly selected from the list of eligible health facilities when possible. More details on the response rate are available in S3 Table in the appendix. All the health facility representatives we managed to reach accepted to take part in the survey.*

S3 Table. Response rate to the health facility phone survey for the round of the study

| Country | Round | # of health facilities selected | # of health facilities interviewed | # of health facilities who cannot be reached or replaced | Response rate |
| --- | --- | --- | --- | --- | --- |
| Bangladesh | Jul-21 | 300 | 291 | 9 | 98% |
| Burkina Faso | Aug-21 | 159 | 159 | 0 | 100% |
| Guatemala | Jun-21 | 255 | 239 | 16 | 94% |
| Guinea | Jul-21 | 160 | 156 | 4 | 98% |
| Liberia | Jul-21 | 122 | 116 | 6 | 97% |
| Malawi | Jun-21 | 204 | 192 | 12 | 94% |
| Nigeria | May-21 | 421 | 401 | 20 | 95% |

**3. What were the criteria used for choosing the countries used in the survey For instance, Only Bangladesh features from SE Asia. The authors must define what were the criteria used for the same**

Response: This study was part of a broader initiative supported by the Global Financing Facility for Women, Children, and Adolescents (GFF) to help member countries to monitor the effect of the COVID-19 pandemic on essential health services. All GFF member countries were offered to participate in the survey. The countries covered in this article were the countries for which at least one round of data was collected by August 2021.

We added the following extract to the overview and sample selection paragraph in the method section to document that point:

*“In this context, implementation of facility phone surveys was offered to all partner countries. The seven countries covered by this study are the ones that opted to implement the phone survey and for which at least one round of data was completed by August 2021.”*

**4. The results are not adequate or complete to draw a proper conclusion from this study. The authors have stated that availability of the PPE does not guarantee usage of PPE by the health care facility, a conclusion which is not adequately supported by the results of the study.**

Response: As we highlight in our paper, the availability of all the PPE barriers at the health facility does not guarantee that HCWs will wear all the recommended items when examining patients suspected to be infected with COVID-19. However, while not sufficient, the availability of all barrier PPE is necessary to comply with infection prevention control protocols.

We believe that our paper makes that point by stressing that, when restricting the sample to health facilities with all PPE barriers available, only 61% of the respondents declared their HCWs were wearing the complete PPE set when examining potential COVID-19 cases.

The results of our study imply that there is not only an urgent need to improve the availability of PPE barriers, as emphasized in the first part the article, but there is also a need for additional interventions to ensure HCWs appropriately use the barriers when they are available.

To make this point clearer we rephrase the following sentences in the result and discussion sections.

Rephrased sentences the result section:

“We also found that HCWs did not use all the recommended PPE barriers when examining COVID-19 suspected or confirmed cases even when all items are available at the health facility. Restricting the sample to only health facilities with the complete PPE set available, we found that HCWs were wearing all the recommended barriers in only 61% of health facilities.”

Rephrased sentence the discussion section:

“We also found that HCWs examining COVID-19 suspected cases were not systematically using the complete PPE set even when all barriers were available in their facilities.”
